# Low Levels of Serum Paraoxonase Activities are Characteristic of Metabolic Syndrome and May Influence the Metabolic-Syndrome-Related Risk of Coronary Artery Disease

**DOI:** 10.1155/2012/231502

**Published:** 2011-09-22

**Authors:** Nicola Martinelli, Roberta Micaglio, Letizia Consoli, Patrizia Guarini, Elisa Grison, Francesca Pizzolo, Simonetta Friso, Elisabetta Trabetti, Pier Franco Pignatti, Roberto Corrocher, Oliviero Olivieri, Domenico Girelli

**Affiliations:** ^1^Department of Medicine, University of Verona, Policlinico G.B. Rossi, 37134 Verona, Italy; ^2^Department of Life and Reproduction Sciences, University of Verona, 37134 Verona, Italy

## Abstract

Low concentrations of plasma high-density lipoprotein (HDLs) are characteristic in metabolic syndrome (MS). The antioxidant ability of HDLs is, at least in part, attributable to pleiotropic serum paraoxonase (PON1). Different PON1 activities have been assessed in 293 subjects with (*n* = 88) or without MS (*n* = 205) and with (*n* = 195) or without (*n* = 98) angiographically proven coronary artery disease (CAD). MS subjects had low PON1 activities, with a progressively decreasing trend by increasing the number of MS abnormalities. The activity versus 7-O-diethyl phosphoryl,3-cyano,4-methyl,7-hydroxycoumarin (DEPCyMC), which is considered a surrogate marker of PON1 concentration, showed the most significant association with MS, independently of both HDL and apolipoprotein A-I levels. Subjects with MS and low DEPCyMCase activity had the highest CAD risk (OR 4.34 with 95% CI 1.44–13.10), while no significant increase of risk was found among those with MS but high DEPCyMCase activity (OR 1.45 with 95% CI 0.47–4.46). Our results suggest that low PON1 concentrations are typical in MS and may modulate the MS-related risk of CAD.

## 1. Introduction

Metabolic syndrome (MS) defines a well-known cluster of metabolic disturbances associated with an increased risk of cardiovascular disease and diabetes [1–3]. Insulin resistance is thought to be the core of MS [2, 3]. Nonetheless, oxidative stress pathways have been also proposed to play a role in MS. An increased oxidative stress, as well as a reduction of antioxidant defences, may impair insulin signalling, therefore leading *per se* to insulin resistance [4]. Moreover, the crucial role of oxidative damage is well documented in both endothelial dysfunction and atherosclerosis processes as it is also accepted that the imbalance of reduction-oxidation (redox) homeostasis may contribute to the development of cardiovascular diseases in MS [4, 5].

Low levels of high-density lipoprotein (HDL) are typical of the biochemical cluster defining MS. HDLs are one of the most important antioxidant defence systems in plasma. They are well known to prevent low-density lipoprotein (LDL) oxidation and protect against LDL-induced cytotoxicity [6–9]. HDLs also possess anti-inflammatory properties, including the ability of suppressing cytokine-induced endothelial cell adhesion molecules function [10–12]. The antioxidant properties of HDLs are, at least to some extent, attributable to serum paraoxonase (PON1). PON1 is a 45 kDa, 355-amino-acid glycoprotein which is synthesized essentially by the liver and then secreted into the blood, where it links to HDLs [13, 14]. PON1 is a pleiotropic enzyme, whose name originally derives from its capacity to neutralize highly toxic, xenobiotic compounds, such as paraoxon. The physiological substrates of PON1, however, are yet partially undefined, even if convincing evidence points to a principal role of PON1 as lactonase, with lipophylic lactones as the primary substrates [15–17]. Previous studies by measuring the rate of paraoxon hydrolysis have shown that subjects with MS had lower PON1 activity [18, 19]. However, it is not clear to what extent the classical, but not physiological, assay, such as the paraoxonase activity, reflects the actual antioxidant capacity of the enzyme. Recently, novel PON1 assays have been developed: 5-thiobutyl butyrolactone (TBBL) and 7-O-diethyl phosphoryl 3-cyano 4-methyl 7-hydroxycoumarin (DEPCyMC) [20]. Both TBBLase and DEPCyMCase activities in sera are highly specific to PON1. TBBL is a chromogenic lactone that resembles the most favourable PON1 lactone substrates, allowing a specific evaluation of PON1 lactonase activity. The DEPCyMC is a chromogenic phosphotriester substrate that, differently from the other methods for PON1 activities, allows estimating total PON1 concentration, the DEPCyMCase assay being not influenced by the degree of catalytic stimulus by HDL. The ratio between these two activities (TBBL-to-DEPCyMC activity ratio) has been suggested to provide the so-called “normalized lactonase activity” (NLA), which may reflect the level of PON1 lactonase catalytic stimulation by HDL [20]. In a previous work, we showed for the first time that these novel PON1 activity assays may be associated with coronary artery disease (CAD). More precisely, we observed that CAD patients had low PON1 concentration, defined by low DEPCyMCase activity, but presented high stimulation of PON1 lactonase activity, as indicated by high NLA values [21].

The aim of this study was to evaluate PON1 activities by means of both traditional (i.e., paraoxonase and arylesterase activity) and new assays (i.e., TBBLase and DEPCyMCase activity) in a population of subjects with or without angiographically confirmed CAD, according to either the presence or absence of MS diagnosis. The potential interaction between MS and PON1 activity as a determinant of CAD risk was also assessed.

## 2. Materials and Methods

### 2.1. Study Population

This study was performed within the framework of the Verona Heart Project, a regional survey designed for identification of new risk factors for CAD in subjects with objective angiographic documentation of their coronary vessels. Details about enrolment criteria have been described in detail elsewhere [22]. As previously reported, a total of 300 subjects were selected and divided into three subgroups matched for sex and age: CAD-free, CAD without myocardial infarction (MI), and CAD with MI [21]. CAD-free (*n* = 100) group had completely normal coronary arteries, being submitted to coronary angiography for reasons other than CAD, mainly valvular heart disease. These controls were also required to have neither history nor clinical or instrumental evidence of atherosclerosis in vascular districts beyond the coronary bed. Two-hundred subjects had angiographically proven CAD with at least one of the main epicardial coronary arteries affected (left anterior descending, circumflex, or right) with ≥1 significant stenosis (≥50%). CAD patients were classified into MI (*n* = 100) and non-MI (*n* = 100) subgroups on the basis of a thorough review of medical records including history, electrocardiogram, enzyme changes, and/or the typical sequelae of MI on ventricular angiography. The angiograms were assessed by cardiologists who were unaware that the patients were to be included in the study. All participants came from the same geographical area (Northern Italy). At the time of blood sampling, a complete clinical history was collected, including the assessment of cardiovascular risk factors such as obesity, smoking, hypertension, and diabetes. From these 300 subjects we further selected 293 subjects (195 CAD and 98 CAD-free) for whom complete data for MS diagnosis were available. According to the established criteria [23], patients were classified as having MS when at least three of the following conditions were present: body mass index >30 kg/m^2^; documented history of hypertension or blood pressure >140/90 mmHg; fasting glucose >6.1 mmol/L; plasma triglycerides >1.7 mmol/L; HDL <1.03 mmol/L for males or <1.29 mmol/L for females.

The study was approved by the Ethic Committee of our Institution (Azienda Ospedaliera, Verona). A written informed consent was obtained from all the participants after a full explanation of the study.

### 2.2. Biochemical Analysis

Samples of venous blood were withdrawn from each subject, after an overnight fast. Serum lipids and the other common biochemical parameters were determined by routine methods. Apolipoprotein A-I (Apo A-I) and Apolipoprotein B (Apo B) were measured by commercially available nephelometric immunoassays; antisera, calibrators, and the BNII nephelometer were from Dade Behring [21]. LDL cholesterol/Apo B ratio was calculated as surrogate marker of small and dense LDL particles [19].

### 2.3. PON1 Activity Assays

PON1 activity assays were performed as previously described [20]. TBBL and DEPCyMC were kindly provided by Dan Tawfik (Department of Biological Chemistry, Weizmann Institute of Science, Rehovot, Israel).

Lactonase activity was measured in activity buffer (50 mM Tris pH 8.0, 1 mM CaCl_2_) containing 0.25 mM of TBBL and 0.5 mM 5,5′-dithio-bis-2-nitrobenzoic acid (DTNB) by monitoring the absorbance at 412 nm in a final volume of 200 *μ*L (*€* = 7,000 OD/M), using an automated microplate reader. The serum was diluted 400-fold in 100 *μ*L of activity buffer complemented with 1 mM DTNB. DTNB was used from 100 mM stock in DMSO. TBBL was used from 250 mM stock in acetonitrile. TBBL was diluted 500-fold in activity buffer containing 2% acetonitrile. The reaction was initiated by adding 100 *μ*L of TBBL (0.5 mM) to 100 *μ*L of sera dilution. The final sera dilution was 800-fold. All the reaction mixtures contained a final 1% acetonitrile. Rates of spontaneous hydrolysis of TBBL in buffer were subtracted from all the measurements. Activities were expressed as U/mL (1 unit = 1 *μ*mol of TBBL hydrolyzed per minute per 1 mL of undiluted serum). 

Total PON1 concentrations in human sera were assessed by measuring the activity with DEPCyMC. For the enzymatic measurements, DEPCyMC was used from 100 mM stock in DMSO, and all the reaction mixtures contained a final 1% DMSO. The activity was measured with 10 *μ*L of serum and 1 mM substrate in 50 mM bis-tris-propane, pH 9.0, with 1 mM CaCl_2_, by monitoring the absorbance at 400 nm in a final volume of 200 *μ*L (*€* = 22,240 OD/M). Activities were expressed as mU/mL (1 milliunit = 1 nmol of DEPCyMC hydrolyzed per minute per 1 mL of undiluted serum).

The normalized lactonase activity (NLA) was calculated by dividing TBBLase activity of each sample by its DEPCyMC activity. 

Paraoxonase activity in sera samples was measured in activity buffer with 1mM paraoxon by monitoring the absorbance at 405 nm (*€* = 10,515 OD/M). Arylesterase activity was measured in activity buffer with 1 mM phenyl acetate by monitoring the absorbance at 270 nm (*€* = 700 OD/M). Activities were expressed as U/L for paraoxon and kU/L for phenyl acetate (1 unit = 1 nmol of paraoxon or 1 *μ*mol of phenyl acetate hydrolyzed per minute per 1 mL of undiluted serum).

All the measures of PON1 activity were performed in duplicate, and all the coefficients of variations were less than 5%.

### 2.4. PON1 Gln_192_Arg and Leu_55_Met Polymorphism Analysis

Genomic DNA was extracted from whole-blood samples by a phenol-chloroform procedure, and subjects were genotyped according to a previously described multilocus assay [24]. PON1 genotypes were available for 264/293 (90.1%) subjects.

### 2.5. Statistics

Calculations were performed with SPSS 17.0 statistical package (SPSS Inc., Chicago, Ill). Distributions of continuous variables in groups were expressed as means ± standard deviation. Statistical analysis on skewed variables, like paraoxonase activity, was computed on the corresponding log-transformed values. However, for the sake of clarity, nontransformed data are reported in the results. Quantitative data were assessed using Student's *t*-test or analysis of variance (ANOVA). Correlations between quantitative variables were assessed using Pearson's correlation test. Qualitative data were analyzed with the *χ* ^2^-test and with *χ* ^2^ for linear trend analysis when indicated. A value of *P* < 0.05 was considered statistically significant. Statistical power was estimated by means of Altmann nomogram.

Within each group examined, the frequencies of the genotypes associated with each of the polymorphisms were compared by the *χ* ^2^-test with the values predicted on the basis of the Hardy-Weinberg equilibrium.

The strength of association of PON1 activity with MS was evaluated by including all the four assessed activities in a multiple regression model with a forward-stepwise variable selection that lastly was adjusted also for HDL and Apo A-I concentrations. The combined effect of MS and DEPCyMCase activity in determining CAD risk was estimated calculating the odds ratios with 95% CIs by multiple logistic regression after adjustment for traditional cardiovascular risk factors not included in MS cluster (i.e., age, sex, smoke, and LDL cholesterol). Finally, the correlation between DEPCyMCase activity and LDL cholesterol/Apo B ratio was evaluated by means of a linear regression analysis model adjusted for age, sex, and HDL concentration.

## 3. Results and Discussion

### 3.1. Results

The clinical characteristics of the study population divided on the basis of MS diagnosis are reported in Table 1. Subjects with (*n* = 88) or without MS (*n* = 205) differed for several characteristics, not only related to MS cluster. As expected, MS was more represented among CAD patients. Moreover, MS subjects presented a lower LDL cholesterol/Apo B ratio. All the four investigated PON1 activities (TBBLase, DEPCyMCase, arylesterase, and paraoxonase activities) were significantly lower in MS subjects, while no significant difference was found for NLA, nor for PON1 genotypes distribution, which respected the Hardy-Weinberg equilibrium (Table 1). No significant difference was found between CAD subjects with or without MI for PON1 activities nor for MS distribution (data not shown).

 Ranking the study population on the basis of MS abnormalities, PON1 activity levels, as well as HDL and Apo A-I concentrations, and LDL-cholesterol/Apo B ratio decreased progressively by increasing the number of metabolic disturbances (Table 2). In particular, DEPCyMCase activity presented a very high significant association with both MS diagnosis (20.60 ± 6.05  versus 23.8 ± 5.6 mU/mL in subjects with or without MS, resp., *P* = 1.58 × 10^−5^) and the number of metabolic disturbances (*P* for linear trend = 3.84 × 10^−6^). Including all the PON1 activities in a regression model with a forward-stepwise variable selection, only DEPCyMCase activity remained a significant predictor of MS (OR for 1 mU/mL increase = 0.90 with 95% CI 0.86–0.95; *P* < 0.001). Noteworthy, this association was independent of HDL and Apo A-I levels (OR for 1 mU/mL increase = 0.93 with 95% CI 0.88–0.98; *P* = 0.004) and of both PON1 genotypes (OR for 1 mU/mL increase = 0.92 with 95% CI 0.86–0.99; *P* = 0.017).

**Figure 1:**
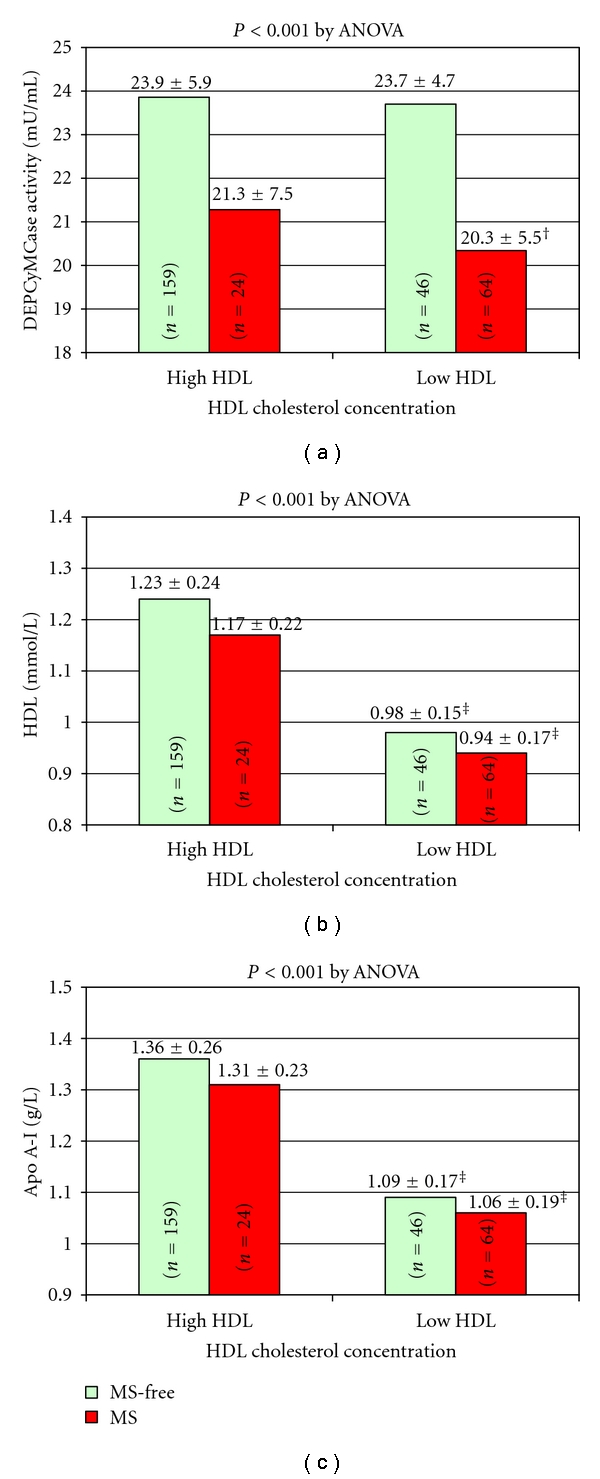
DEPCyMCase activity (a), HDL cholesterol (b), and apolipoprotein A-I concentration (c) according to high/low HDL cholesterol levels and metabolic syndrome (MS) diagnosis (b). Low HDL-cholesterol concentrations are defined on the basis of ATP-III criteria for MS-diagnosis, that is, <1.03 mmol/L for males or <1.29 mmol/L for females. ^†^Significantly lower than no-MS with high or low HDL (*P* < 0.01 by Tukey post-hoc comparison). ^‡^Significantly lower than high HDL with or without MS (*P* < 0.01 by Tukey post-hoc comparison).

Consistently with the last outcome, by stratifying the study population on the basis of MS diagnosis and HDL levels, which were considered as a dichotomic variable on the basis of MS-related threshold level, subjects with low HDL levels but without MS had a higher DEPCyMCase activity than subjects with equally low HDL levels and MS (Figure 1(a)). Interestingly, subjects with low HDL levels but without MS presented a DEPCyMCase activity comparable with that of subjects with high HDL levels and without MS (Figure 1(a)). On the other hand, subjects with high HDL levels but with MS showed a trend versus lower DEPCyMCase activity compared to those with high HDL levels and without MS, even if this difference did not reach statistical significance. Noteworthy, no difference in HDL or Apo A-I concentration appeared to justify such results in the above-mentioned four subgroups. Indeed, within the groups with either low or high HDL levels, MS diagnosis was not associated with additional differences in HDL and Apo A-I concentrations (Figures 1(b) and 1(c)), thus supporting an HDL-independent, Apo A-I-independent association of DEPCyMCase activity with MS.

**Figure 2:**
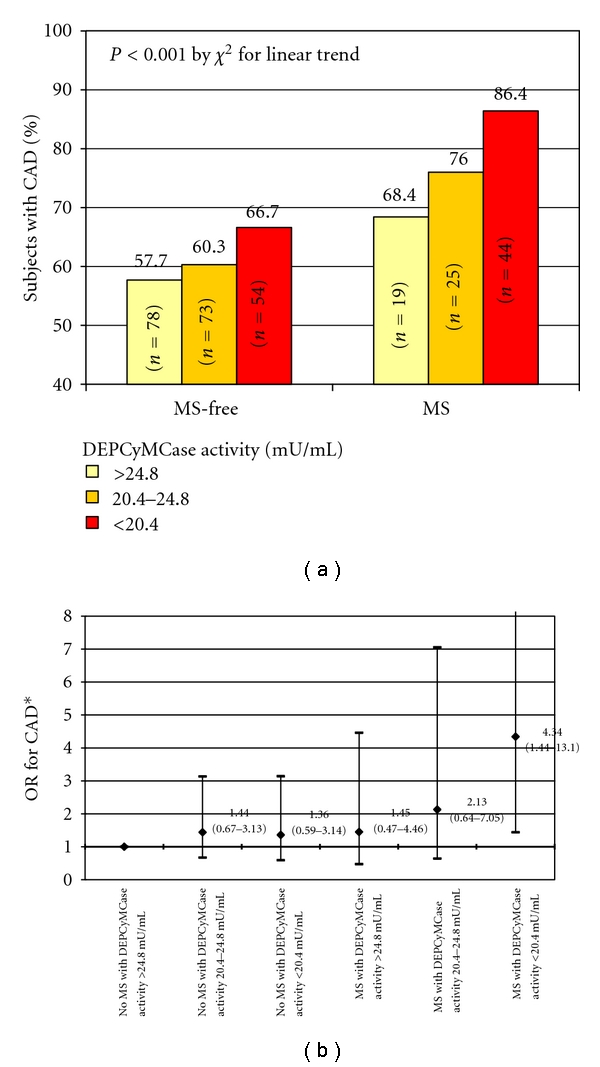
Prevalence of subjects with coronary artery disease (CAD) according to metabolic syndrome (MS) diagnosis and DEPCyMCase activity (a) and the relative ORs for CAD in a multiple adjusted regression model considering subjects without MS and within the highest DEPCyMCaseactivity tertile as reference group (b). *B y multiple logistic regression adjusted for classical CAD risk factors not included in MS definition, that is, sex, age, smoke and LDL cholesterol.

Considering that MS is linked to an increased risk of CAD and that an association of low DEPCyMCase activity with CAD has been previously found in the same study population [21], an analysis about potential combined effects on CAD risk was performed. In a multiple logistic regression model, both MS and DEPCyMCase activity remained significantly associated with CAD (OR for MS = 2.17 with 95% CI 1.19–3.97, *P* = 0.012; OR for 1 mU/mL increase of DEPCyMCase activity = 0.95 with 95% CI 0.91–0.99, *P* = 0.033). After stratifying the study population on the basis of MS diagnosis and DEPCyMCase activity tertiles, the statistical analysis highlighted a progressive increase of CAD diagnosis prevalence among subjects without MS but within the highest DEPCyMCase activity tertile group, compared to those with MS and within the lowest DEPCyMCase activity tertile (Figure 2(a)). Considering subjects without MS and with the highest DEPCyMCase activity tertile as reference group, subjects with MS and low DEPCyMCase activity presented a marked increase of CAD risk, even after adjustments for the classical cardiovascular risk factors not included in MS cluster (OR 4.34 with 95% CI 1.44–13.10, Figure 2(b)), while no significant increase of CAD risk was found for those with MS but high DEPCyMCase activity (OR 1.45 with 95% CI 0.47–4.46). Such results were confirmed also by including PON1 genotypes in the regression model (for subjects with MS and low DEPCyMCase activity: OR 4.67 with 95% CI 1.12–15.15).

**Figure 3:**
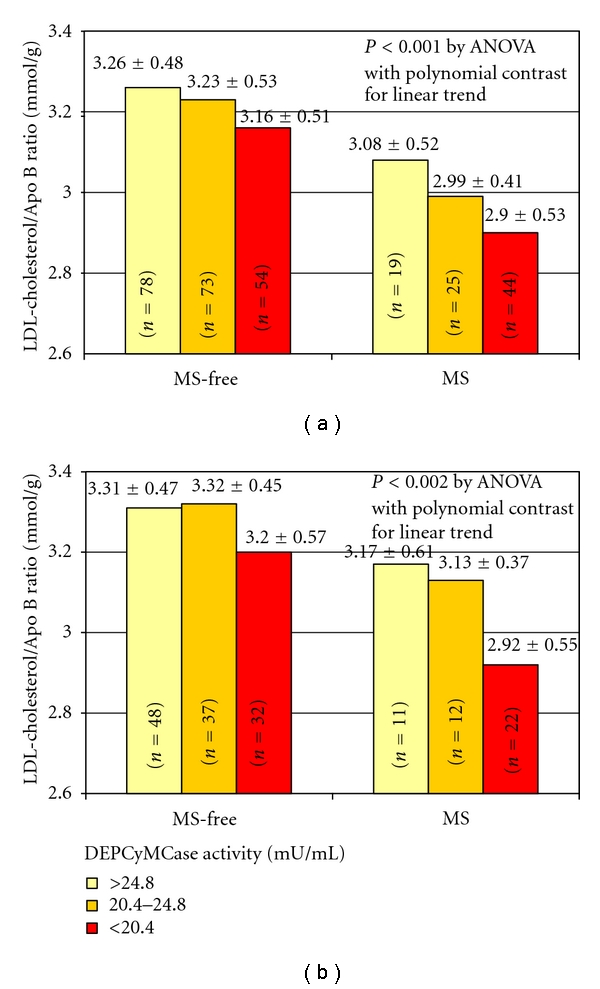
LDL cholesterol/Apo B ratio according to metabolic syndrome (MS) diagnosis and DEPCyMCase activity in the whole population (a) (*n* = 293) and in subjects without lipid-lowering therapy (b) (*n* = 162).

An analysis on LDL cholesterol/Apo B ratio was also performed. The LDL cholesterol/Apo B ratio was used as a surrogate marker for small and dense LDLs, where the lower ratios suggested a higher prevalence of this type of LDL particles. A low LDL cholesterol/Apo B ratio was associated with both CAD (3.09 ± 0.57 versus 3.36 ± 0.60 mmol/g in CAD-free, *P* < 0.001) and MS (3.00 ± 0.57 versus 3.26 ± 0.59 mmol/g in MS-free, *P* = 0.001). Furthermore, the ratio progressively decreased with the consensual increase of the number of metabolic disturbances (Table 2). DEPCyMCase activity showed also a positive correlation with LDL cholesterol/Apo B ratio (Pearsons'coefficient 0.165, *P* = 0.008) that remained significant even after a regression model adjusted for age, gender, and HDL levels (standardized beta-coefficient 0.158, *P* = 0.014) was performed. By stratifying the study population on the basis of MS diagnosis and DEPCyMCase activity tertiles, there was a progressive decrease of LDL cholesterol/Apo B ratio from subjects without MS and within the highest DEPCyMCase activity tertile toward those subjects who instead had MS and were within the lowest DEPCyMCase activity tertile. These findings were observed both when the analysis was performed in the whole study population (Figure 3(a)) and even after the exclusion of subjects taking lipid-lowering therapy (Figure 3(b)).

## 4. Discussion

The current study, consistently with some previous observations [18, 19], shows a substantial impairment of PON1 activities in patients affected by MS.

MS is characterized by a constellation of metabolic abnormalities that altogether lead to an increased risk of cardiovascular diseases. Although there is a substantial disagreement over the terminology and diagnostic criteria of MS and even some controversies exist about whether MS is a true syndrome or a mixture of various phenotypes [1–3], it is undeniable that the clustering of MS abnormalities occurs jointly more often than by chance alone, suggesting the possibility of an underlying, common pathogenesis [1]. A biologically plausible hypothesis proposes that MS presents when an excess of body fat accumulates in subjects with a specific metabolic susceptibility which seems likely represented by insulin resistance [2]. On the other hand, MS is also known to be associated with a prooxidant and proinflammatory status. Moreover, oxidative stress is considered to play a pivotal role in MS pathophysiology, favouring atherosclerotic damage and increasing CAD risk [4].

PON1 is a HDL-associated, pleiotropic enzyme and may play a role in several different pathways: from the protection against oxidative damage and lipid peroxidation to the contribution to innate immunity processes and from the detoxification of reactive molecules and/or xenobiotic compounds to drug bioactivation (e.g., clopidogrel) [14, 25–27]. More specifically, PON1 is capable of protecting lipoproteins from lipid peroxidation by degrading specific oxidized cholesteryl esters and phospholipids, and antioxidant properties of HDL have been attributed, at least partially, to PON1 [25, 28, 29]. On the other hand, PON1 can be, in turn, inactivated by oxidative stress and oxidized lipids [30]. Thus, there are many biologically plausible reasons linking the pro-oxidant, HDL-poor MS with the anti-oxidant, HDL-associated PON1.

In the first study linking PON1 and MS, Senti and coworkers observed a progressive decrease of paraoxonase activity by increasing the number of MS disturbances [18]. Concomitantly, a progressive increase of lipid peroxides concentration was observed, so that the authors hypothesized that a greater degree of severity of MS is associated with an increased oxidative stress which inactivates PON1 function. On the other hand, they emphasized also the possibility that low PON1 function fails an efficient protection against MS-related oxidative damage that cannot be excluded [18]. Later on, those results were confirmed by Blatter Garin and colleagues, who found a low paraoxonase activity and a reduced PON1 mass in patients with MS [19]. These authors also found that MS patients had a decreased LDL cholesterol/Apo B ratio, indicative of the presence of small, dense, oxidized, and proatherogenic lipoprotein particles [19].

Our study extends such findings also for the novel PON1 assays utilized, the DEPCyMCase and TBBLase activity. Moreover, the most significant association found in our analysis was that with DEPCyMCase activity, which is considered the best surrogate marker of PON1 concentration, since, differently from the other assay to test PON1 activities, it is not influenced by PON1 genotype nor is it stimulated by HDL binding [20].

Although it could be argued that MS-related, low PON1 concentration is merely an epiphenomenon of low level of HDLs (i.e., the plasma carriers of PON1), our analysis does not support this point of view, the association of DEPCyMCase activity with MS being independent of both HDL and Apo A-I levels. This HDL-independent association is further emphasized by observing that subjects with low HDL level but without MS appeared to have a “normal” not reduced DEPCyMCase activity, while those with high HDL level but with MS tended to have a reduced DEPCyMCase activity (Figure 1(a)). Taking all together, these results suggest that a low DEPCyMCase activity, that is, a low PON1 concentration, is characteristic of MS, independently of low HDL concentration. Moreover, they invite to take into account not only HDL quantity but also HDL quality, which could be reflected, at least in part, by PON1 concentration/activity. It is noted, in this regard, that subjects with low HDL but without MS may have a small amount of “high-quality” HDLs, while subjects with high HDL levels but with MS may have a large amount of “low-quality” HDLs. The relevance of considering HDL quality and not only its quantity has been indirectly emphasized by the failure of the cholesteryl ester transfer protein (CETP) inhibitor, torcetrapib, to improve cardiovascular clinical end points. It has been in fact reported that torcetrapib, in spite of its demonstrated efficiency to elevate HDL concentration, eventually leads instead to an increased cardiovascular morbidity, even if also other off-target adverse effects for CAD risk (e.g., blood pressure elevation) have been related to torcetrapib use [31, 32]. However, in any case, these results have underscored the intricacy of HDL metabolism, with functional quality perhaps being more important than the circulating quantity of HDL [33, 34]. Indeed, both quantitative and qualitative changes to lipoprotein profiles may lead to an increased CAD risk and PON1 could be considered as a potential marker of HDL quality, being linked to the antioxidant, anti-inflammatory properties of HDL.

Accordingly with this last consideration, in our study PON1 appears to influence the MS-related risk of CAD. Certainly, an additive effect between MS and PON1 levels (marked by DEPCyMCase activity) should be considered in determining CAD risk, which increased progressively from subjects without MS and with high PON1 levels to those with MS and low PON1 levels. Remarkably, subjects with MS but still high PON1 levels did not present a significant increase of CAD risk. This result is consistent with a previous study showing that an increased expression of human PON1 in a mouse model of MS inhibited the development of atherosclerosis, probably by reducing the amount of oxidized LDL in both plasma and atherosclerotic plaque [35]. Moreover, it is worthy of note that, in our study population, there was a significant, positive correlation between DEPCyMCase activity and LDL cholesterol/Apo B ratio, suggestive of high PON1 levels associated with low levels of small, dense, and oxidized LDL. Remarkably, LDL cholesterol/Apo B ratio trend across the MS-DEPCyMCase activity stratification groups (Figure 3) was impressively consistent with that of CAD risk (Figure 2). Taken together these results, we are tempting to speculate that an adequate function of PON1 may counterbalance some of MS-related, harmful effects at vascular level, probably by reducing oxidative stress and lipid peroxidation, and thus protect against MS-associated risk of CAD. If this hypothesis is true, by restoring PON1 function, there could be a possibility for a new therapeutic tool for cardiovascular diseases, with particular advantageous effects in high-risk patients, such subjects with MS. However, PON1 reduction may be merely the result of a more extensive PON1 consumption/inactivation during conditions characterized by increasing severity of oxidative stress, like a more advanced MS [19] or a clinically evident CAD. Therefore, further studies are needed to address the issue whether PON1 reduction could be a pathogenic, contributory factor to increased risk of CAD in MS or that reduction would be simply the consequence of ancillary enzyme consumption/inactivation due to CAD/MS-associated oxidative stress.

### 4.1. Study Limitations

There are some significant caveats to the present study. The retrospective case-control design, the small number of enrolled subjects, and the lack of some clinical data, such as the waist circumference value, are possible limitations of this study. However, despite the relatively low sample size of our study population, the statistical power of the analysis for DEPCyMCase activity difference between MS and MS-free subjects was >90% by Altmann nomogram with a significance level at 0.05. As further experimental limitation, it should be underlined that the DEPCyMCase activity is only a surrogate marker of PON1 concentration and that we did not perform a direct PON1 quantification by ELISA. However, in a previous study the DEPCyMCase assay has been shown to provide information similar to PON1 ELISA assay [20]. Also LDL cholesterol/Apo B ratio is regarded as a surrogate marker of small and dense LDL and, in addition, Apo B was measured in whole serum, although it is known to essentially reflect Apo B in LDL. On the other hand, a remarkable strength of this study is represented by the angiography evaluation of the coronary artery bed, which allows a clear-cut definition of the clinical phenotype and avoids the possibility to include in the control group subjects with subclinical, but significant CAD.

## 5. Conclusions

In summary, our results show that PON1 activities, including those evaluated by means of novel substrate assays (TBBL and DEPCyMC), are impaired in MS. In particular, these assays suggest that a low PON1 concentration, as indicated by the low DEPCyMCase activity, is characteristic of MS cluster, independently of HDL concentration. Moreover, DEPCyMCase activity appears to interact with MS in determining the risk of CAD, with data suggesting that this association may be also related to the prevalence of small, dense, and oxidized LDL. More precisely, a high DEPCyMCase activity (i.e., a high PON1 concentration) seems protective for CAD risk in subjects with MS, while the highest risk for CAD was observed among subjects with MS and concomitant low levels of DEPCyMCase activity (i.e., a low PON1 concentration). Certainly, a statistically significant association does not mean a link of causality. Nonetheless, PON1 assays seem to add more important information than the simple HDL quantity assessment. According to this hypothesis of heterogeneous HDL-related effects, a recent study demonstrated that in contrast to HDL from healthy subjects, HDL from patients with CAD does not have endothelial anti-inflammatory effects and does not stimulate endothelial repair because it fails to induce endothelial NO production [36]. Mechanistically, the reduction of HDL-associated PON1 activity appears to lead to inhibition of eNOS activation and the subsequent loss of the endothelial antiinflammatory and endothelial repair-stimulating effects of HDL [36], thus supporting the concept that the cardiovascular impact of HDL is not simply related to its abundance [37]. Indeed, HDL cholesterol is only an integrative but nonfunctional measure of lipoproteins, and, therefore, novel biomarkers reflecting the functionality of HDL particles are needed to assess and better monitor cardiovascular risk [34], in particular in the presence of conditions at high CAD risk such as the case of MS. PON1 assays could be potentially considered as such new diagnostic tools while further studies are needed to address this intriguing issue.

## Figures and Tables

**Table 1:** Characteristics of the study population, with or without metabolic syndrome (MS).

Characteristics	MS-free (*n* = 205)	MS (*n* = 88)	*P*
Age (years)	60.9 ± 9.3	60.8 ± 9.6	NS*
Male sex (%)	59.5	42.0	0.006^†^
Coronary Artery Disease (%)	61.0	79.5	0.002^†^
BMI (kg/m²)	25.5 ± 2.9	28.7 ± 5.1	<0.001*
Hypertension (%)	57.1	89.8	<0.001^†^
Smoking (%)	52.8	54.9	NS ^†^
Diabetes (%)	7.8	46.5	<0.001^†^
Glucose (mmol/L)	5.40 ± 0.98	7.01 ± 2.29	<0.001*
Creatinine (*µ*mol/L)	90.1 ± 64.2	89.4 ± 21.9	NS*
Total cholesterol (mmol/L)	5.16 ± 1.13	5.29 ± 1.19	NS*
LDL-cholesterol (mmol/L)	3.18 ± 0.95	3.33 ± 1.11	NS*
HDL-cholesterol (mmol/L)	1.35 ± 0.34	1.07 ± 0.31	<0.001*
Triglycerides (mmol/L)	1.47 ± 0.65	2.33 ± 0.98	<0.001*
Apo A-I (g/L)	1.30 ± 0.26	1.13 ± 0.23	<0.001*
Apo B (g/L)	0.98 ± 0.24	1.08 ± 0.28	0.003*
TBBLase activity (U/mL)	3.45 ± 0.98	3.02 ± 1.02	0.001*
DEPCyMCase activity (mU/mL)	23.83 ± 5.65	20.60 ± 6.05	<0.001*
Paraoxonase activity (U/L)	120.8 ± 79.1	102.6 ± 71.5	0.030*
Arylesterase activity (kU/L)	101.3 ± 31.4	86.8 ± 32.5	<0.001*
Normalized lactonase activity	145.0 ± 24.3	146.1 ± 35.0	NS*

PON1 Gln_192_Arg ^‡^			
Gln/Gln	50.3	45.6	
Gln/Arg	40.0	49.4	NS^†^
Arg/Arg	9.7	5.1	*

PON1 Leu_55_Met^‡^			
Leu/Leu	38.0	38.0	
Leu/Met	48.9	44.3	NS^†^
Met/Met	13.1	17.7	

*by *t*-test; ^†^by *χ*² test; ^‡^PON1 genotype data were available for 264/293 (90.1%) subjects, that is, 185 MS-free and 79 with MS; NS: no significant.

**Table 2:** Serum paraoxonase (PON1) activities, HDL cholesterol, and apolipoprotein A-I (Apo A-I) concentrations and LDL cholesterol/Apo B ratio according to the number of metabolic syndrome (MS) abnormalities.

	0 MS element (*n* = 35)	1 MS element (*n* = 78)	2 MS elements (*n* = 92)	3 MS elements (*n* = 50)	4 MS elements (*n* = 31)	5 MS elements (*n* = 7)	P*
TBBLase activity (U/mL)	3.60 ± 1.38	3.46 ± 0.95	3.38 ± 0.80	3.08 ± 0.99	2.98 ± 1.13	2.69 ± 0.56	<0.001*

DEPCyMCase activity (mU/mL)	24.49 ± 7.62	23.97 ± 5.90	23.45 ± 4.49	21.74 ± 5.96	19.36 ± 6.23	17.92 ± 4.37	<0.001

Paraoxonase activity (U/L)	128.4 ± 103.7	118.8 ± 69.2	119.5 ± 77.1	102.2 ± 63.0	103.6 ± 89.7	101.4 ± 38.3	NS

Arylesterase activity (kU/L)	106.3 ± 35.7	101.3 ± 31.1	99.3 ± 30.0	89.1 ± 34.0	85.6 ± 32.3	76.0 ± 21.0	<0.001

Normalized Lactonase Activity	145.4 ± 23.3	145.3 ± 24.4	144.7 ± 24.9	140.8 ± 31.9	152.8 ± 39.1	154.3 ± 34.7	NS

HDL cholesterol (mmol/L)	1.25 ± 0.26	1.19 ± 0.25	1.14 ± 0.25	1.05 ± 0.23	0.97 ± 0.15	0.83 ± 0.19	<0.001

Apo A-I(g/L)	1.38 ± 0.25	1.31 ± 0.27	1.27 ± 0.26	1.18 ± 0.25	1.09 ± 0.17	0.93 ± 0.21	<0.001

LDL cholesterol-to-Apo B ratio (mmol/g)	3.32 ± 0.43	3.31 ± 0.73	3.21 ± 0.65	3.04 ± 0.65	2.97 ± 0.45	2.88 ± 0.57	0.001

*by ANOVA with polynomial contrast for linear trend.
